# LncRNA MEG3 targeting miR-424-5p via MAPK signaling pathway mediates neuronal apoptosis in ischemic stroke

**DOI:** 10.18632/aging.102790

**Published:** 2020-02-16

**Authors:** Yanxiao Xiang, Yayun Zhang, Yanni Xia, Hua Zhao, Anchang Liu, Yuguo Chen

**Affiliations:** 1 Department of Pharmacy, Qilu Hospital of Shandong University, Jinan 250012, Shandong, China; 2 School of Medicine, Shandong University, Jinan 250100, Shandong, China; 3 Department of Emergency Medicine and Chest Pain Center, Qilu Hospital of Shandong University, Jinan 250012, Shandong, China; 4 Department of Orthopedics, Qilu Hospital of Shandong University, Jinan 250012, Shandong, China; 5 Department of Operating Room, Qilu Hospital of Shandong University, Jinan 250012, Shandong, China; 6 Clinical Research Center for Emergency and Critical Care Medicine of Shandong Province, Institute of Emergency and Critical Care Medicine of Shandong University, Qilu Hospital of Shandong University, Jinan 250012, Shandong, China; 7 Key Laboratory of Emergency and Critical Care Medicine of Shandong Province, Key Laboratory of Cardiopulmonary-Cerebral Resuscitation of Shandong Province, Qilu Hospital of Shandong University, Jinan 250012, Shandong, China; 8 The Key Laboratory of Cardiovascular Remodeling and Function Research, Chinese Ministry of Education, Chinese Ministry of Health and Chinese Academy of Medical Sciences, The State and Shandong Province Joint Key Laboratory of Translational Cardiovascular Medicine, Qilu Hospital of Shandong University, Jinan 250012, Shandong, China

**Keywords:** ischemic stroke, MEG3, miR-424-5p, *Sema3A*

## Abstract

Emerging evidence suggests that long non-coding RNAs (lncRNAs) are significant regulators in the pathological process of ischemic stroke (IS). However, little is known about lncRNAs and their roles in IS. In this study, we aimed to screen out differentially expressed lncRNAs and revealed the underlying mechanisms in IS. The results of bioinformatic analysis showed that lncRNA MEG3 and *Sema3A* were over-expressed in IS samples, while miR-424-5p was lower-expressed. Correlation between MEG3/miR-424-5p, and miR-424-5p/*Sema3A* were predicted with miRanda and TargetScan, and verified by dual luciferase assay. Inhibition of MEG3 remarkably increased the expression of miR-424-5p and decreased the expression of *Sema3A*, which also led to in an increased cell viability and decreased cellular apoptosis in oxygen-glucose deprivation and reoxygenation (OGD/R) model, as well as an activated MAPK signaling pathways. Consistently, MEG3 was upregulated in MCAO mice, knockdown of MEG3 reduced the infarct volume and improved neurobehavioral outcomes in rats following MCAO. In conclusion, it was demonstrated that MEG3 accelerated the process of IS by suppressing miR-424-5p, which targeted *Sema3A* and the activated MAPK pathway. These results might provide useful information for exploring the potential therapeutic targets in IS.

## INTRODUCTION

Ischemic stroke (IS) is the main cause of disability and mortality above the age of 60, and accounts for about 87% of all the strokes [1]. Although older people bear a higher risk of stroke, there is a dramatic increase of incidence rate in people aged 25 to 44, which might result from various factors like hypertension, hypercholesterolemia, diabetes, smoking and obesity [2]. Traditional clinical management of stroke includes thrombolytic therapy, percutaneous intravascular interventions, behavioral rehabilitation strategies, and medication such as aspirin. However, the limitations include a narrow time window or expertise demand. [3]. Luckily, advancements in genetic engineering in recent years have promoted the understanding of many biological molecules in IS, among which lncRNA appealed to researchers’ interest.

LncRNAs are non-protein coding RNA molecules longer than 200 nucleotides (nt) [4]. They can regulate chromosomes to alter the gene expression in transcription, or serve as a competing endogenous RNA (ceRNA) at post-transcriptional level [1]. The roles of some lncRNAs in IS have been identified by Dykstra et al. recently. Microarray or RNA-seq analysis was used to discover hundreds of differentially expressed lncRNAs in IS patients [5]. For all those discovered lncRNAs, we focus on MEG3, which is short for human and mouse maternally expressed gene 3 and is also known as gene trap locus 2 (Gtl2) in mouse. It is widely expressed in brain and many other cells. Many researches showed that MEG3 was a tumor suppressor in colorectal cancer [6] and other cancers [7]. In IS, Yan et al. reported that ischemia altered cerebral MEG3 profiles *in vitro* and *in vivo* [8]. They also found that MEG3 functioned as a competing endogenous RNA to regulate ischemic neuronal death [9]. As it has been proved that lncRNA and miRNA interact with each other in various diseases including several tumors and ischemic disorders [10, 11], we further hypothesized that MEG3 function as a ceRNA competitively binding to miRNAs in the development of ischemia.

Similar to lncRNAs, miRNAs are also non-protein coding but consist of 18 to 22 nt [12]. Apart from the interaction with lncRNAs mentioned above, miRNAs could affect the stability of mRNAs, subsequently decrease the downstream protein production [13]. Many studies disclosed the function of miRNAs in various biological processes, including cell growth, differentiation, apoptosis, development and homeostasis [14, 15]. The change of their expression could cause various diseases including cancer [16]. As for IS, circulating miRNAs like, miR-125a-5p, miR-125b-5p and miR-143-3p are differentially expressed after acute IS, and their expression levels are correlated with infarct volume and stroke etiology [17]. MiR-424 was reported to suppress microglia activation and thus protects against permanent focal cerebral ischemic injury in mice [18, 19]. Besides, miR-424-5p has been found to participate in the regulation of cellular activities *via* interacting with specific lncRNA sponges [20]. Therefore, we sought to further find out how miR-424-5p mediated IS progression.

The downstream mRNA of miR-424-5p focused in this study was *Sema3A*, a member of the Semaphorins family implicated in angiogenesis, construction of nerve network and tumor development [21]. *Sema3A* was found to be associated with many different signaling pathways. The collapse of growth cone induced by *Sema3A* was mediated partly by activating ERK as well as p38 MAPK [22]. Meanwhile, the activation of *Sema3A*/Cdc42/JNK pathway might lead to dysregulation of a series of apoptosis-related regulators and subsequently promotes apoptosis [23]. The localization of GSK-3β in axons induced by inhibition of *Sema3A* through Rnd1/R-Ras/Akt/GSK-3β pathway was found to be beneficial to the outgrowth of axons of cortical neurons which result in a better prognosis [24]. Although the association between *Sema3A* and MAPK signaling has been reported, the inner mechanism remains poorly understood. To figure out the mechanism, we mainly researched the effect of *Sema3A* on IS through MAPK signaling pathway.

In this study, the impacts of MEG3 on IS progress was investigated to clarify what function MEG3 serves in IS pathology. In addition, to further understand the molecular mechanism, the regulatory relationship between MEG3/miR-424-5p/*Sema3A* axis and downstream MAPK signaling pathway was validated both *in vitro* and *in vivo*. Our results revealed that MEG3 and *Sema3A* were up-regulated in IS, while miR-424-5p was down-regulated. Inhibition of MEG3 protected against cerebral ischemic insults. These finding indicated that the MEG3/miR-424-5p/*Sema3A* axis could be valuable for the development of novel IS detective, preventive and therapeutic methods.

## RESULTS

### *Sema3A* was overexpressed in IS

**Figure 1:**
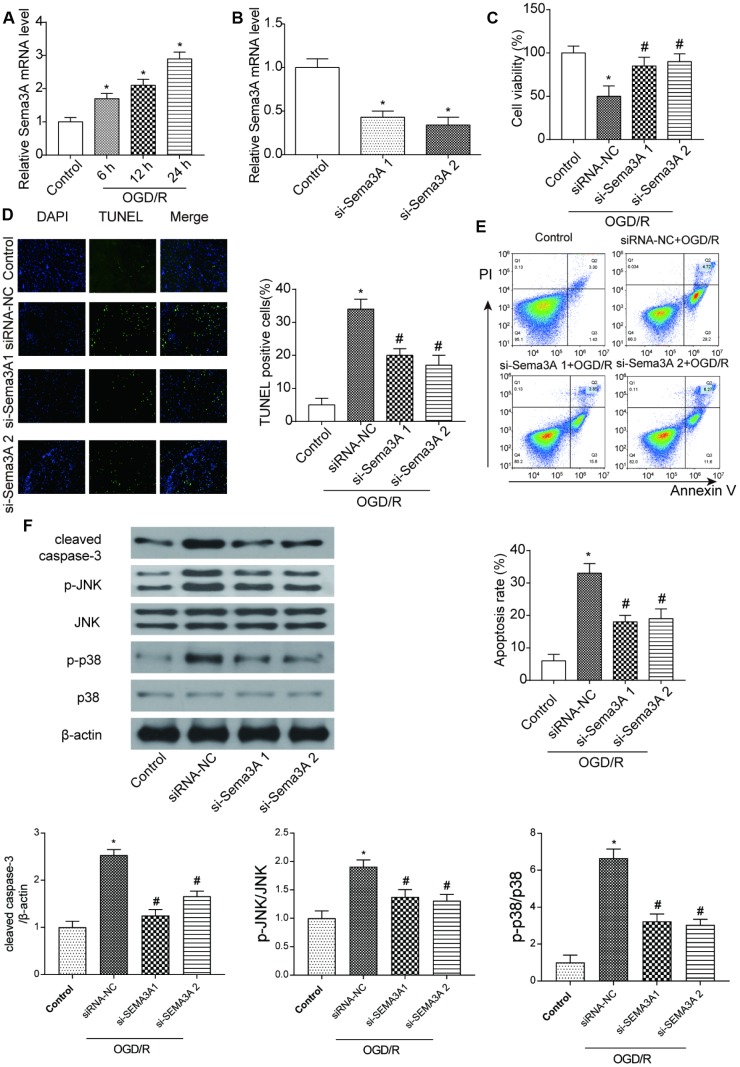
**MRNA *Sema3A* was highly expressed in IS and correlated with cell viability and apoptosis.** (**A**) The mRNA level of *Sema3A* in N2a cells climbed up with time after 6h, 12h, 24h of OGD/R treatment. *P<0.05 vs control. (**B**) Successful downregulation of *Sema3A* by si-*Sema3A*. *P<0.05 vs control. (**C**) *Sema3A* downregulation led to higher viability of OGD/R cells. *P<0.05 vs control, #*P*<0.05 vs siRNA-NC. (**D**, **E**) OGD/R cells have higher apoptosis rate compared with control groups while *Sema3A* downregulation is correlated with lower apoptosis level among OGD/R cells. *P<0.05 vs control, #*P*<0.05 vs siRNA-NC. (**F**) OGD/R cells tend to have higher level of c-caspase-3 as well as p-JNK and p-p38 in comparison with control group. Downregulation of *Sema3A* could counteract with this trend in OGD/R cells partly. *P<0.05 vs control, #*P*<0.05 vs siRNA-NC.

The heatmap was obtained by microarray analysis of GSE22255, the results were listed in Supplementary Table 1. Supplementary Figure 1A showed the top 10 differentially upregulated and downregulated mRNAs in IS. We screened out a variety of signaling pathways upregulated in control group and IS group (Supplementary Figure 1B). The significantly activated or suppressed pathways were represented by the dotplot and ridgeplot, and MAPK signaling pathway was one of the evidently suppressed pathways in IS (Supplementary Figure 1C, 1D). *Sema3A* along with *DUSP1* and *FOS* were recognized as the top 3 aberrantly expressed molecules related to MAPK signal pathway (Supplementary Figure 1E). Later, we treated N2a cells with OGD/R to simulate IS *in vitro*. These 3 molecules were collected for qRT-PCR to cross-check the abnormal expression in control and OGD/R cell lines. *DUSP1* and *FOS* displayed no significant difference in OGD/R group (Supplementary Figure 1F) while *Sema3A* expression rose over time of intervention (Figure 1A).

### *Sema3A* mediated cell viability and apoptosis in OGD/R cells

To detect the function of *Sema3A*, we conducted a series of experiments by manually altering the expression of *Sema3A*. N2a cells used in the following experiments were under 2h OGD treatment and cultured under normal conditions for 24 h. It was demonstrated that *Sema3A* was successfully downregulated in the OGD/R cells by si-*Sema3A* (Figure 1B). In si-*Sema3A* groups, cell viability was notably higher than that in the control group (Figure 1C). TUNEL staining and flow cytometry assay both showed that downregulation of *Sema3A* led to less cell apoptosis of OGD cells (Figure 1D–1E). However, expression levels of phosphorylated JNK and p38 decreased after knockdown of *Sema3A*, indicating that *Sema3A* had an activating effect on the MAPK signaling pathway (Figure 1F).

### MiR-424-5p was lower-expressed in IS and targeted *Sema3A*

**Figure 2:**
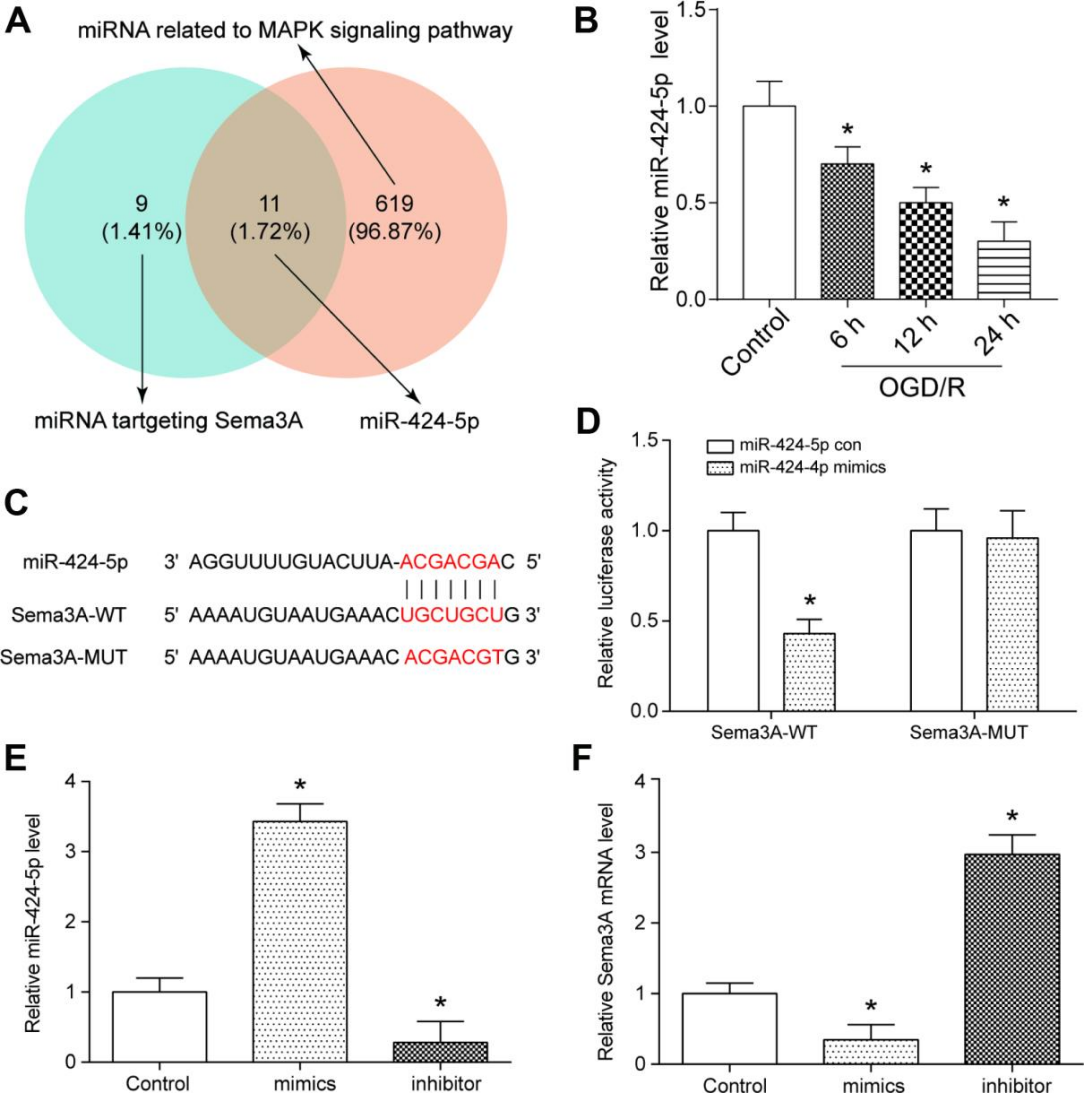
**MiR-424-5p targeted with *Sema3A* and was lowly expressed in IS.** (**A**) MiR-424-5p was among 11 miRNAs which both targeted *Sema3A* and related to MAPK signaling pathway. (**B**) The relative miR-424-5p expression declined with time of 6h, 12h, 24h of OGD/R treatment. *P<0.05 vs control. (**C**, **D**) The target relationship between *Sema3A* and miR-424-5p was predicted by Targetscan and verified by dual luciferase assay. *P<0.05 vs control. (**E**, **F**) Upregulation of miR-424-5p by miR-424-5p mimics led to lower *Sema3A* expression, while downregulation of miR-424-5p led to higher *Sema3A* expression. *P<0.05 vs control.

We used TargetScan to predict *Sema3A*-related miRNAs and used DIANA TOOLS to verify miRNAs related to MAPK signaling pathway. As one of the common miRNAs related to *Sema3A* and MAPK signaling pathway, miR-424-5p was screened out by using Venny 2.1 (Figure 2A). The expression level of miR-424-5p in control and OGD/R group was detected by qRT-PCR. In contrast with control group, miR-424-5p expression demonstrated a decline along with the prolonged duration of treatment. (Figure 2B). TargetScan was used to predict the binding position between *Sema3A* and miR-424-5p (Figure 2C), and dual luciferase assay was applied to verify the interactive relationship (Figure 2D). We also tested the *Sema3A* expression after manually modifying the expression of miR-424-5p. MiR-424-5p upregulation by miR-424-5p mimics led to downregulation of *Sema3A*, while miR-424-5p downregulation by miR-424-5p inhibitor could promote *Sema3A* expression (Figure 2E, 2F). Hence, the expression of *Sema3A* could be regulated by miR-424-5p.

### MEG3 was highly expressed in IS and interacted with miR-424-5p

**Figure 3:**
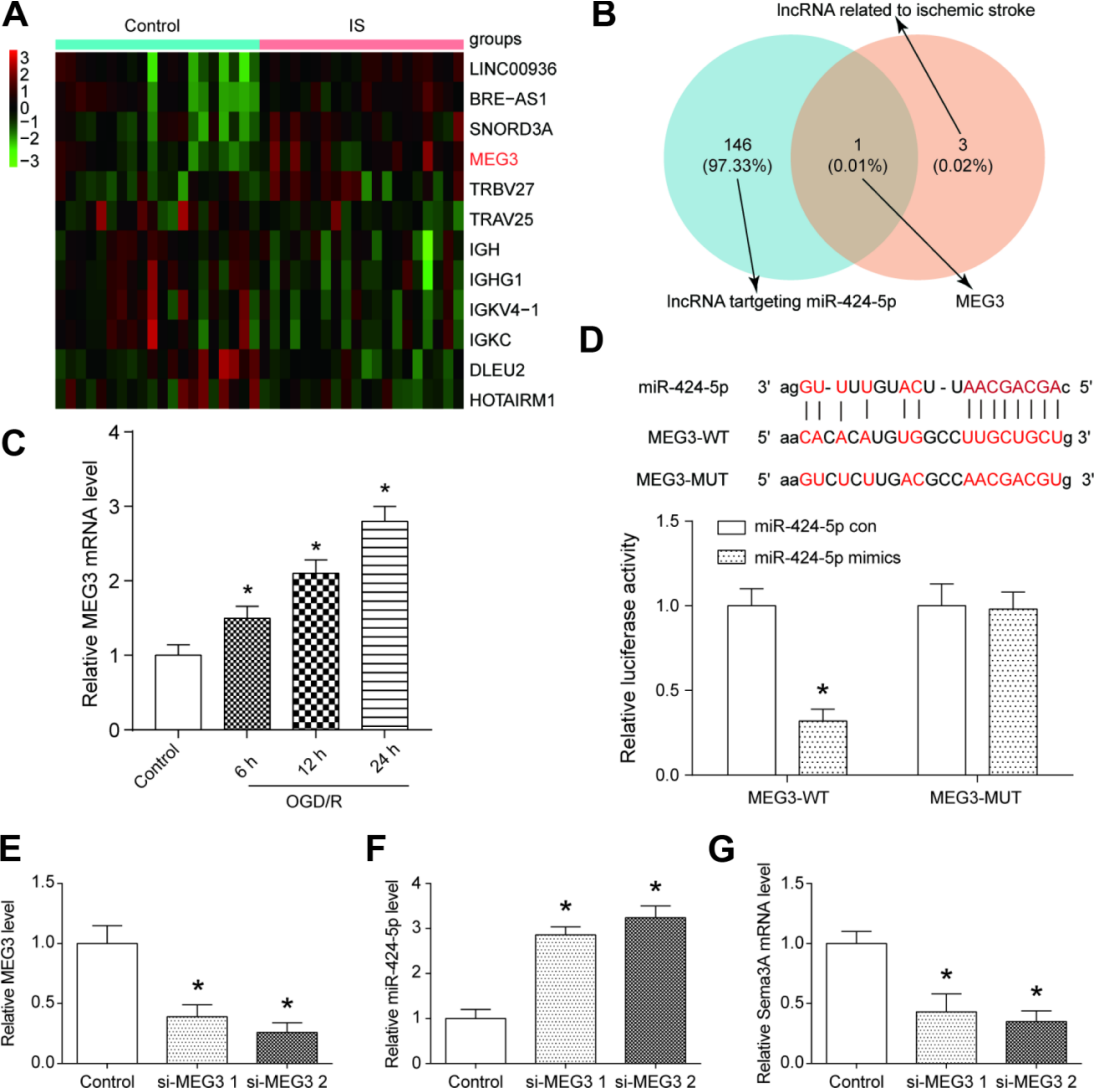
**MEG3 was a highly expressed lncRNA in IS that bonded with miR-424-5p.** (**A**) The heatmap from the microarray analysis of GSE22255 showed the significantly differentially expressed lncRNAs including MEG3 in the IS group. (**B**) MEG3 bound to miR-424-5p and was related to IS. (**C**) MEG3 expression was positively correlated with OGD/R intervention time. *P<0.05 vs control. (**D**) The target relationship between MEG3 and miR-424-5p was predicted and proved. *P<0.05 vs control (**E**) The effect of downregulation MEG3 by si-MEG3 was proved significant. *P<0.05 vs control. (**F**) MEG3 knockdown correlated with higher miR-424-5p expression. *P<0.05 vs control. (**G**) MEG3 knockdown correlated with lower *Sema3A* expression. *P<0.05 vs control.

We further analyzed the differentially expressed lncRNAs in GSE22255 (Supplementary Table 2), and disclosed that MEG3 was one of the most significantly overexpressed lncRNAs in IS group (Figure 3A). With the help of the MiRanda and LncRNA and Disease Database, we found that MEG3 is both related to miR-424-5p and IS (Figure 3B). In N2a cells, the expression of MEG3 increased along with OGD treatment time, and this trend could also be observed on the expression of *Sema3A* (Figure 3C). The binding position between MEG3 and miR-424-5p was predicted by MiRanda and the target relationship was verified by dual luciferase assay (Figure 3D). Forced downregulation of MEG3 by si-MEG3 led to a higher expression level of miR-424-5p and a lower expression level of *Sema3A* (Figure 3E–3G). All of these results revealed that MEG3 could regulate *Sema3A* expression by targeting miR-424-5p.

### MEG3 inhibited cell viability and promoted cell apoptosis by positively regulating *Sema3A*

**Figure 4:**
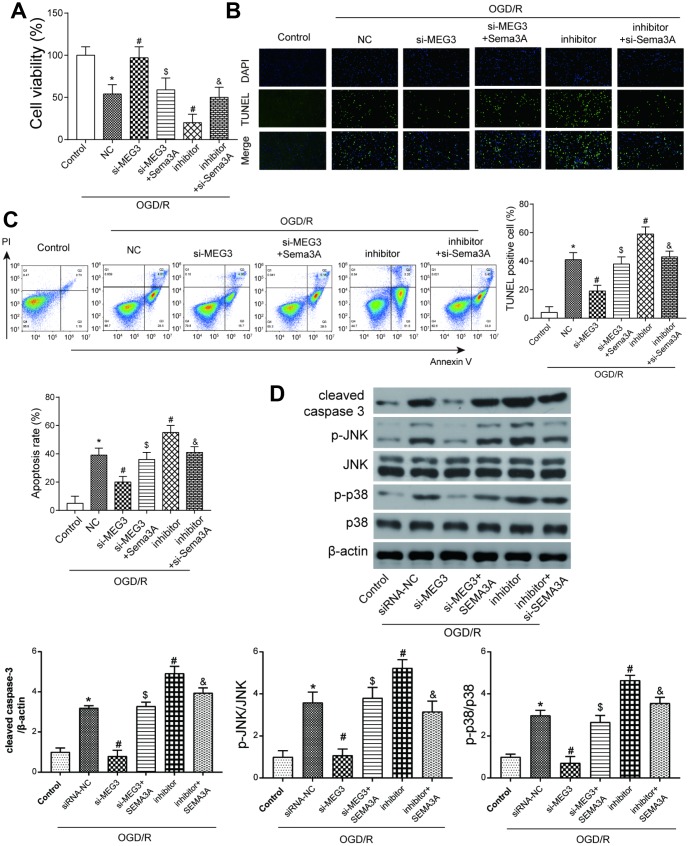
**The MEG3/miR-424-5p/*Sema3A* axis affected cell viability and apoptosis in OGD cells via the MAPK pathway.** (**A**) OGD/R cells have lower cell viability compared with control group. Among OGD/R cells, MEG3 downregulation led to higher cell viability, which could be offset by a simultaneous overexpression of *Sema3A*; miR-424-5p downregulation by its inhibitor led to a lower cell viability compared among OGD/R cells, which could be neutralized by a simultaneous downregulation of *Sema3A*. *P<0.05 vs control, #*P*<0.05 vs OGD/R NC, $*P*<0.05 vs si-MEG3, &*P*<0.05 vs inhibitor (**B**, **C**) OGD/R cells tend to have fairly high apoptosis rate compared to control group. Among OGD/R cells, MEG3 downregulation led to lower cell apoptosis level, which could be neutralized by a simultaneous overexpression of *Sema3A*; miR-424-5p downregulation by its inhibitor mediated more cell apoptosis, which could be neutralized by a simultaneous downregulation of *Sema3A*. *P<0.05 vs control, #*P*<0.05 vs OGD/R NC, $*P*<0.05 vs si-MEG3, &*P*<0.05 vs inhibitor. (**D**) OGD/R cells tend to have higher cleaved caspase-3, p-JNK and p-p38 expression in comparison with control. Among OGD/R cells, the low expression of MEG3 led to a rise in expression of cleaved caspase-3, p-JNK and p-p38, miR-424-5p inhibitor led to drop of expression of cleaved caspase-3, p-JNK and p-p38. *Sema3A* overexpression could reverse the rise of si-MEG3, while si-*Sema3A* could reverse the drop of miR-424-5p inhibitor. *P<0.05 vs control, #*P*<0.05 vs OGD/R NC, $*P*<0.05 vs si-MEG3, &*P*<0.05 vs inhibitor.

To further verify the effect of potential MEG3/miR-424-5p/*Sema3A*/MAPK axis on the development of IS, a series of experiments detecting cell viability, apoptosis and MAPK signal pathway were conducted. In OGD/R-treated cells, cell viability decreased compared to the control group. si-MEG3 group where MEG3 expression was down-regulated showed recovered viability while a co-transfection of *Sema3A* could impair this restoration. On the contrary, miR-424-5p inhibitor led to a lower cell viability, which could be reversed by co-transfection of si-*Sema3A* (Figure 4A). According to TUNEL staining and flow cytometry results, apoptosis rate of OGD/R cells increased dramatically compared to the control group. In OGD/R treated groups, the downregulation of MEG3 restrained apoptosis, while miR-424-5p inhibitor promoted it. The effect of down-regulation of MEG3 and miR-424-5p could be reversed partially by co-transfection of *Sema3A* and si-*Sema3A* respectively (Figure 4B, 4C). To observe alteration in the downstream MAPK signal pathway, the level of cleaved caspase-3 and phosphorylated levels of JNK and p38 were detected through western blot. All of these markers displayed similar trends among different groups. OGD/R cells displayed higher cleaved caspase-3/caspase-3 ratio as well as p-JNK/JNK and p-p38/p38 ratio indicating enhanced apoptosis and activated MAPK signal pathway. This trend could be partially counteracted through down-regulation of MEG3 and reversed by co-transfection of *Sema3A*. Meanwhile, inhibition of miR-424-5p could promote apoptosis and MAPK signal pathway while co-transfection of si-*Sema3A* could help to counteract it (Figure 4D). These results further indicate that MEG3 activated the MAPK signaling pathway through regulating miR-424-5p/*Sema3A* axis.

### MEG3 expression aggravated severity of IS by promoting *Sema3A* expression *in vivo*

**Figure 5:**
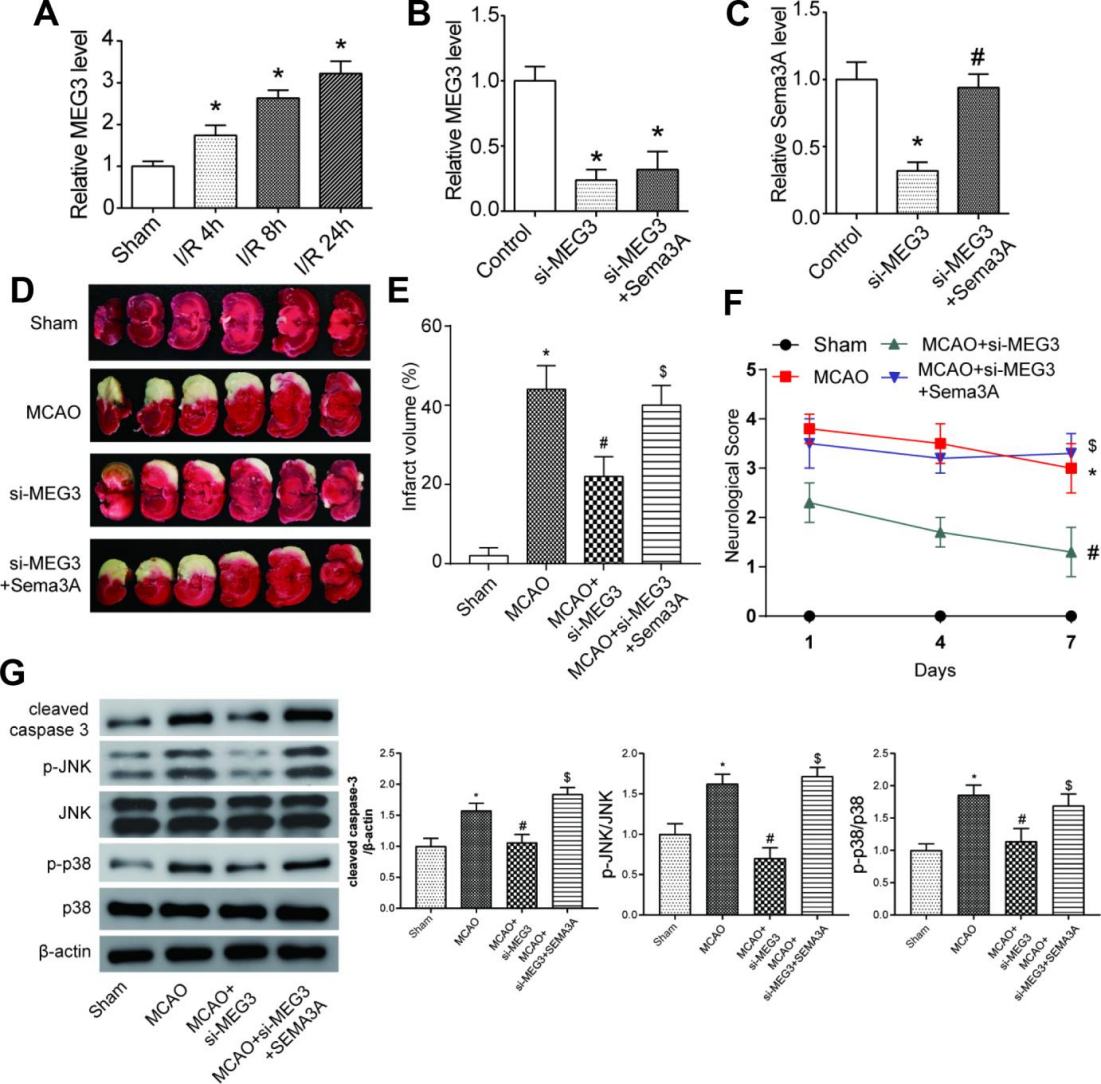
**MEG3 expression accelerated the process of IS by promoting *Sema3A* expression *in vivo*.** (**A**) Expression level of MEG3 increased gradually at 4h, 8h, 24h point in the process of ischemia reperfusion (I/R stands for ischemic reperfusion). *P<0.05 vs sham. (**B**) Downregulation of MEG3 expression by si-MEG3 was proved significant. Simultaneous upregulation of *Sema3A* had little influence on the MEG3 expression. *P<0.05 vs control. (**C**) *Sema3A* expression was suppressed by si-MEG3, and the decrease was neutralized by manual *Sema3A* overexpression. *P<0.05 vs control, #*P*<0.05 vs si-MEG3. (**D**) Representative image of brain slices of three groups of rats. Normal tissues were a pink or red color, whereas the ischemic tissues were white. MCAO rats tend to have a bigger ischemic area in contrast with Sham group. Among MCAO rats, si-MEG3 group with MEG3 down-regulated have smaller ischemic area while it could be reversed by overexpression of *Sema3A*. (**E**) Quantified average percentage of the infarction area in the whole brain, the percentage was higher in MCAO rats compared with sham group. Among MCAO rats, the infarct volume decreased in si-MEG3 group, while simultaneous overexpression of *Sema3A* weakened the decrease. *P<0.05 vs sham, #*P*<0.05 vs MCAO, $*P*<0.05 vs MCAO+si-MEG3. (**F**) Neurological score rose in MCAO rats compared with sham rats. Among MCAO rats, si-MEG3 group had lower neurological score than MCAO control, while simultaneous overexpression of *Sema3A* neutralized the decrease of score. *P<0.05 vs sham, #*P*<0.05 vs MCAO, $*P*<0.05 vs MCAO+si-MEG3. (**G**) Expression of cleaved caspase-3, p-JNK and p-p38 increased significantly in MCAO group compared with control group. In si-MEG3 group, expression of cell apoptosis markers cleaved caspase-3 and phosphorylated levels of JNK and p38 decreased. Overexpression of *Sema3A* reversed the change. *P<0.05 vs sham, #*P*<0.05 vs MCAO, $*P*<0.05 vs MCAO+si-MEG3.

In MCAO mice models, the MEG3 expression level was increased, compared to the sham group (Figure 5A). Before the ischemic reperfusion, si-MEG3 and si-MEG3+*Sema3A* transfection were conducted on mice in experimental groups. Results showed that 72h after the reperfusion, *Sema3A* co-transfection could almost reverse the downregulation of *Sema3A* by si-MEG3, but had no effect on the inhibition of MEG3 in the mice models (Figure 5B, 5C). The infarct area in MCAO group increased dramatically compared with Sham group. In si-MEG3 group, the infarct area of MCAO mice shrank and it could be reversed in the si- MEG3+*Sema3A* group (Figure 5D, 5E). At the same time, the neurological score rose significantly in comparison with sham group. si-MEG3 group could partly offset the increase in neurological score in MCAO mice, while *Sema3A* co-transfection counteracted this effect (Figure 5F). All the results above indicated MEG3 could aggravate IS progress by regulating *Sema3A* expression. According to the western blot results in Figure 5G, the expression of cell apoptosis marker cleaved caspase-3 was increased in MCAO mice in contrast with the control group, so did MAPK signal pathway markers-phosphorylated JNK and p38. This trend in MCAO mice could be offset by down-regulating MEG3, which could then be reversed by the co-transfection of *Sema3A* indicating that MEG3 promoted cell apoptosis and activated MAPK signaling pathway by modulating *Sema3A*.

## DISCUSSION

In this study, we mainly illustrated a mechanism of MEG3/miR-424-5p/*Sema3A* axis mediating ischemic stroke. LncRNA MEG3 mediated the progression of IS by targeting miR-424-5p, which targets *Sema3A*. Knockdown of MEG3 or *Sema3A* led to higher cell viability and less cell apoptosis in OGD/R-treated N2a cells. On the contrary, miR-424-5p over-expression suppressed *Sema3A* expression, which subsequently inhibited cell viability and accelerated cell apoptosis. *In vivo*, inhibition of MEG3 protected against cerebral ischemic insults.

Bioinformatic analysis of the data set GSE22255 screened out an mRNA highly expressed in IS, *Sema3A*, as well as a *Sema3A*-related MAPK signaling pathway, which was aberrantly modulated in IS. As a growth and apoptosis-related gene, the role of *Sema3A* had been explored in traumatic brain injury [25], autoimmune disease [21] and tumors [26]. According to previous studies, *Sema3A* functioned as a negative guidance molecule which led to an inhibitory environment where axonal growth was retarded and growth cone collapse [27]. In addition, *Sema3a* was found to be able to competitively bind to Neuropilin-1 (Nrp-1) which might antagonize the effect of vascular endothelial growth factor (VEGF). This interaction could lead to vascular degradation and prevent the revascularization of the ischemic area which might cause bad prognosis [28]. Over-expressed in injured brain tissues, *Sema3A* was considered as a potent culprit of neuronal death [29] and the inhibition of which reduced apoptosis and resulted in better prognosis [24]. In the present study, we introduced the role of *Sema3A* and provided new potential therapeutic targets of IS by verifying the hidden mechanism with miRNA and lncRNA involved.

We screened out miR-424-5p which targeted *Sema3A* and verified its negative regulation on *Sema3A* expression. MiR-424-5p were found lowly-expressed in IS, and its expression was negatively related to deterioration of IS. Other than IS, miR-424-5p had also been found to be related to various diseases including neuroblastoma [30]. In breast cancer, miR-424-5p functioned as a tumor suppressor, regulating tumor cell proliferation, migration and invasion by binding with the oncogene doublecortin like kinase 1 (*DCLK1*) [31]. Similarly, in epithelial ovarian cancer, miR-424-5p also played a tumor-suppressing role by inhibiting CCNE1 expression and obstructing cell cycle in G1/G0 phase [32]. In our study, we verified the interaction between miR-424-5p and *Sema3A* in IS, and uncovered how miR-424-5p promoted cell viability and suppressed cell apoptosis in IS through *Sema3A*.

The regulatory mechanism underlying MEG3/miR-424-5p/*Sema3A* axis was demonstrated both *in vitro* and *in vivo*. MEG3 was a widely researched lncRNA in neoplasms, including osteosarcoma [33], glioma [34], gallbladder cancer [35], and breast cancer [36]. It has been known that MEG3 bound to its targets by RNA-DNA triplex structures [37]. By analyzing ChRIP-seq data, researchers found that MEG3 interacted with the Polycomb repressive complex 2 (PRC2 complex). The binding sites for MEG3 RNA revealed some of the TGF-β pathway genes as direct targets [37]. The functions of MEG3 varied depending on type of cancer. For example, in breast cancer, up-regulating the expression of MEG3 had a potential to inhibit tumor growth by suppressing miR-21 via the PI3K/Akt signaling pathway [38]. Furthermore, MEG3 regulated the pathogenesis of advanced chronic myeloid leukemia by targeting miR-147 through the JAK/STAT pathway [39]. Another study showed that lower expression of MEG3 was related to poor recurrence-free survival in bladder cancer [40]. In our research, we expanded known knowledge of MEG3 by verifying its role in IS. Our study of the co-effect network of MEG3, miR-424-5p and *Sema3A* revealed the mechanism behind how MEG3 expression promoted cell apoptosis in IS.

In summary, we discovered in the present study that MEG3 regulated cell viability and cell apoptosis in IS by binding with miR-424-5p, which targeted gene *Sema3A* and modulate the MAPK signaling pathway. The MEG3/miR-424-5p/*Sema3A* may be promising effective biomarkers and therapeutic targets of IS.

## MATERIALS AND METHODS

### Microarray analysis

The data set of GSE22255 obtained from The Gene Expression Omnibus (GEO) was analyzed to reveal the differentially expressed genes and activated signaling pathway in IS tissues, and was first published on Dec 31, 2011 by Tiago Krug, et al [41]. This data set includes gene expression profiling of peripheral blood mononuclear cells (PBMCs) collected from 20 IS patients and 20 matched controls. The data were collected 6 months after the onset of stroke and were analyzed using Affymetrix microarrays. Important variables such as age and sex are matched between IS group and control group. Several researches regarding IS arose from this dataset since then.

Differential analysis of mRNAs was performed using "limma' package in R 3.4.1 (https://www.r-project.org/), and mRNAs with |log_2_Fold change|>0.5 and *P*<0.05 were chosen for further analysis.

TargetScan database (www.targetscan.org) was employed to screen out the potential upstream regulator of target effector molecule. In addition, DIANA Tool (http://diana.imis.athena-innovation.gr/DianaTools/index.php) was applied to filter out microRNAs related to the MAPK signaling pathway. The intersection of these 2 sets were used to detect the microRNA for further research.

Once the microRNA was determined, we used MiRanda (http://www.microrna.org/) to predict potential lncRNAs interacting with microRNA mentioned above. Meanwhile, The LncRNA and Disease Database (http://www. cuilab.cn/lncrnadisease) was used to find out the IS associated lncRNAs. The lncRNAs of the intersection of these 2 sets were chosen for further study.

### Gene set enrichment analysis (GSEA) analysis

Total normalized mRNAs expression data were uploaded into GSEA v3.0 software. KEGG pathway gene set was used to explore enriched pathways, and GO term gene set was used to explore enriched functions. We also employed R language “GSEABase” package to conduct data processing. The Benjamini-Hochberg Procedure was used to adjust *P* values. Statistic of default weighted enrichment was employed to manipulate data for 1000 times with normalized *P*<0.05 which was considered significantly enriched. Next, results at the top and on the bottom of GSEA reports were respectively chosen for curves using “ggplot2” package in R language.

### Cell culture

We adopted the mouse brain neuroma cell line, N2a, to build oxygen-glucose deprivation (OGD) model. The cells were purchased from BeNa Culture Collection (Shanghai, China), and maintained in a medium of 90% DMEM (Invitrogen, Carlsbad, CA, USA) and 10% FBS (Invitrogen, Carlsbad, CA, USA), at 37°C in 5% CO_2_.

### Oxygen and glucose deprivation and reoxygenation (OGD/R) model

Cells were washed and then incubated in deoxygenated glucose-free DMEM. The cultures were then transferred to an anaerobic chamber filled with a gas mixture of 95% N_2_/5% CO_2_ at 37°C for 4h. At the end of OGD treatment, the medium was replaced with normal medium, and the cultures were returned to a normal atmosphere for 6, 12 and 24 hours respectively. Control cells were cultured under normoxic conditions without OGD treatment. With this condition, we harvested the 6, 12 and 24 hours of OGD/R cells for the detection of mRNAs, miRNAs and lncRNAs.

### Cell transfection

The overexpression vectors of *Sema3A* constructed by GenePharma (Shanghai, China). MiR-424-5p mimics, inhibitor, MEG3 siRNAs and *Sema3A* siRNAs (listed in Supplementary Table 3) were designed and purchased from Sigma Aldrich (St. Louis, MO, USA). All RNAs were transfected into OGD/R model cells by the Lipofectamine 3000 Transfection Reagent (Invitrogen, Carlsbad, CA, USA) in accordance with the manufacturer’s instructions.

### Quantitative real-time reverse transcription-PCR (RT-PCR)

Total RNAs were extracted from cells and tissues by Trizol (Invitrogen, Carlsbad, CA, USA) according to the protocols. Then we carried out reverse transcription by the PrimeScript^®^ RT Kit (TAKALA, Dalian, China), and performed quantitative PCR in a 7500 Real-Time PCR System (Applied Biosystems, USA) using SYBR^®^ Premix Ex Taq™ II (TAKALA, Dalian, China). The expression of *Sema3A*, miR-424-5p and MEG3 was normalized to the level of GAPDH and U6. We calculated all results using the 2^−ΔΔCT^ method. The experiments were performed three times and the sequences were listed in Supplementary Table 4.

### Cell counting Kit-8 (CCK-8) assay

48h after the transfection, cells were seeded in 96-well plates with a density of 2×10^4^ cells/well and incubated overnight with 5% CO_2_ at 37°C. CCK-8 solution (Beyotime, Shanghai, China) was added (10 μL/well) to each well, and stored at 37°C for an additional 4h. Then, we detected the viability of cells per well through measuring the absorbance at 450nm using Elx800 Reader (Bio-Tek Instruments Inc., Winooski, VT, USA).

### TUNEL staining

Cell apoptosis was determined by means of in situ cell Death Detection kit (Roche, Indianapolis, IN, USA). After the treatment, 0.2% Triton X-100 was added into cells for a 5min incubation. Then, the cells were incubated with the TUNEL reaction mixture containing the nucleotide mixture and terminal deoxynucleotidyl transferase (TdT). Subsequently, the cells were incubated in 0.3% H_2_O_2_ for 10min and stained with 0.5μg/mL DAPI in the dark for 5min at room temperature. TUNEL staining with a green fluorescence showed apoptotic cells, DAPI with a blue fluorescence showed the nuclei. The cells were observed and calculated under a fluorescence microscope (Olymbus, Tokyo, Japan). Five visual fields were chosen randomly for each specimen.

### Flow cytometry

After transfection, OGD/R-treated N2a cells were incubated for 48h before cell apoptosis analysis. Incubated cells were stained with a FITC Annexin V Apoptosis Detection Kit (BD Biosciences, Bedford, MA, USA), and then put through FCM detection.

### Western blot

We lysed the cells or tissues in RIPA protein extraction regent (Beyotime) with a protease inhibitor cocktail provided by Roche Diagnostics, Mannheim, Germany. Protein concentration was measured using BCA protein assay kit form Abcam (Cambridge, MA, USA). The equal amounts of proteins approximately 50μg were separated by 10% SDS-PAGE, and then were transferred to control membrane (Millipore, Billerica, MA, USA). Proteins were blocked with 5% milk at room temperature for 1h, and then incubated with primary antibodies, rabbit monoclonal JNK, p-JNK, p38, p-p38, caspase-3, cleaved caspase-3 and β-actin (anti-JNK, ab179461, 1:1000; anti-p-JNK, ab124956, 1:1000; anti-p38, ab170099, 1:1000; anti-p-p38, ab4822, 1:1000; anti-caspase-3, ab44976, 1:500; anti-cleaved caspase-3, ab2302, 1:1500; anti-β-actin, ab8227, 1:10000, Abcam, Cambridge, MA, USA) at 4°C overnight, and last incubated with Goat Anti-Rabbit IgG H&L (HRP) (ab6721, 1:10000, Abcam) at room temperature for 1h. We analyzed the protein bands by the Pierce ECL Substrate Western Blot Detection system (Bio-Rad Laboratories, Hercules, CA, USA).

### Luciferase reporter assay

We predicted the sequences of wild-type (WT) *Sema3A* and MEG3 containing the targeting position of miR-424-5p. Then the synthesized *Sema3A*-WT, *Sema3A*-MUT, MEG3-WT and MEG3-MUT were cloned into the downstream gene via the dual luciferase reporter vectors (Promega, Madison, WI, USA). The configured luciferase vectors were named as *Sema3A*-WT, *Sema3A*-MUT, MEG3-WT and MEG3-MUT respectively. We co-transfected *Sema3A*-WT and *Sema3A*-MUT as well as MEG3-WT and MEG3-MUT with miR-control or miR-424-5p mimics using Lipofectamine2000 (Invitrogen, Carlsbad, CA, USA). We measured the luciferase activities by Dual Luciferase Assay (Promega, Madison, WI, USA) after transfection 48h.

### Animals

Adult male C57BL/6J mice weighing 22 g -25 g were used for this study. Mice were allowed 1 week for adaption. The mice were arranged into four groups: the sham group, the MCAO group, the MCAO+si-MEG3 (intraventricular injection si-MEG3) group and the MCAO+si-MEG3+*Sema3A* group (intraventricular injection si-MEG3 and *Sema3A*). The methods for intraventricular injection were: i) mice were anesthetized with 10% chloral hydrate (3.2 ml/kg) by intraperitoneal injection and then placed on stereotactic frame; ii) si-MEG3 as well as si-MEG3+*Sema3a* were delivered to the right lateral ventricle through a Hamilton microsyringe (Hamilton Co., Reno, NV, USA); All experiments involving animals were in accordance with the institutional animal welfare guideline and approved by Institutional Animal Care and Use Committee of Qilu Hospital of Shandong University.

### Mice model of middle cerebral artery occlusion (MCAO)

One day after the intraventricular injection of MEG3 siRNA and *Sema3A*, the mice were performed with the operation of MCAO. In brief, after anesthetized with ketamine (ip, 80-100mg/kg) as well as xylazine (ip, 10mg/kg), the right common carotid artery [42], external carotid artery (ECA) and internal carotid artery (ICA) were sequentially isolated. An incision was made in the distal region of the CCA, then a 6-0 nylon monofilament with a round tip was inserted into the ICA until the origin of the MCA was blocked. After 60 minutes of ischemia, the nylon monofilament was softly withdrawn to allow reperfusion. Laser Doppler flow was employed to monitor the blood flow and animals with a blood flow reduction less than 80% were excluded for further experiment. Other vital physical variables such as heart rate, body temperature and blood pressure were monitored through the operation. Sham underwent the same procedure except for the insertion of filament. At 72h after the reperfusion, the brain tissues were collected for TTC staining and expression level of MEG3, miR-424-5p, *Sema3A* and MAPK signaling pathway related proteins were detected through qRT-PCR and western blot. At 1, 3, 5, and 7 days following the reperfusion, the neurological deficits was assessed via a modified Bederson scoring system of five-point scale as described in a previous study [43].

### TTC staining and measurement of infarct volume

Tri-phenyl-tetrazolium chloride (TTC) staining was used to measure cerebral infarction volume 72h after reperfusion. We incubated the 2-mm-thick dissected brain slices with a 2% TTC solution at 37°C for 30 min. Later, TTC-stained slices were photographed using a Nikon E950 digital camera attached to a dissecting microscope and determined the percentage of the infarct volume in the total brain volume in the digitized images with the Quantity One software package (Bio-Rad, Hercules, CA, USA). Normal tissues showed a pink or red color, and ischemic tissues were white.

### Neurological score system

The modified Bederson scoring system [43] was used to quantify the neurological behaviors. We trained rats for 3 consecutive days before surgery, and used the score on the day before MCAO as the baseline. The test was performed at 1, 4 and 7 days following the reperfusion. A score of 0 meant no deficit; 1 for forelimb flexion; 2 for unidirectional circling after lifted by tail; 3 for spontaneous unidirectional circling; 4 for longitudinal rolling upon lifted by tail and 5 for spontaneous longitudinal rolling. Consequently, average score in each group reflected severity of neurological deficit - higher score meant severer deficit.

### Statistical analysis

All the measurement data were expressed with mean ± standard deviation [44] and visualized by GraphPad Prism 6.0. We compared the difference between every two groups of data by *t*-test. One-way ANOVA was used to compare the differences among multiple groups. We repeated all experiments for at least three times, with *P*<0.05 indicating that the difference was significant statistically.

### Ethical approval

All procedures performed in studies involving animals were in accordance with the ethical standards of Qilu Hospital of Shandong University. This article does not contain any studies with human participants performed by any of the authors.

## Supplementary Material

Supplementary Figure 1

Supplementary Tables
