# Supplementary material for: LncRNA MEG3 targeting miR-424-5p via MAPK signaling pathway mediates neuronal apoptosis in ischemic stroke
Source: Aging (Albany NY). 2020 Feb 16;12(4):3156–74. doi: 10.18632/aging.102790 (PMC7066902; doi:10.18632/aging.102790)
Supplement: Supplementary Figure 1 [file aging-12-102790-s002..pdf]

SUPPLEMENTARY FIGURE

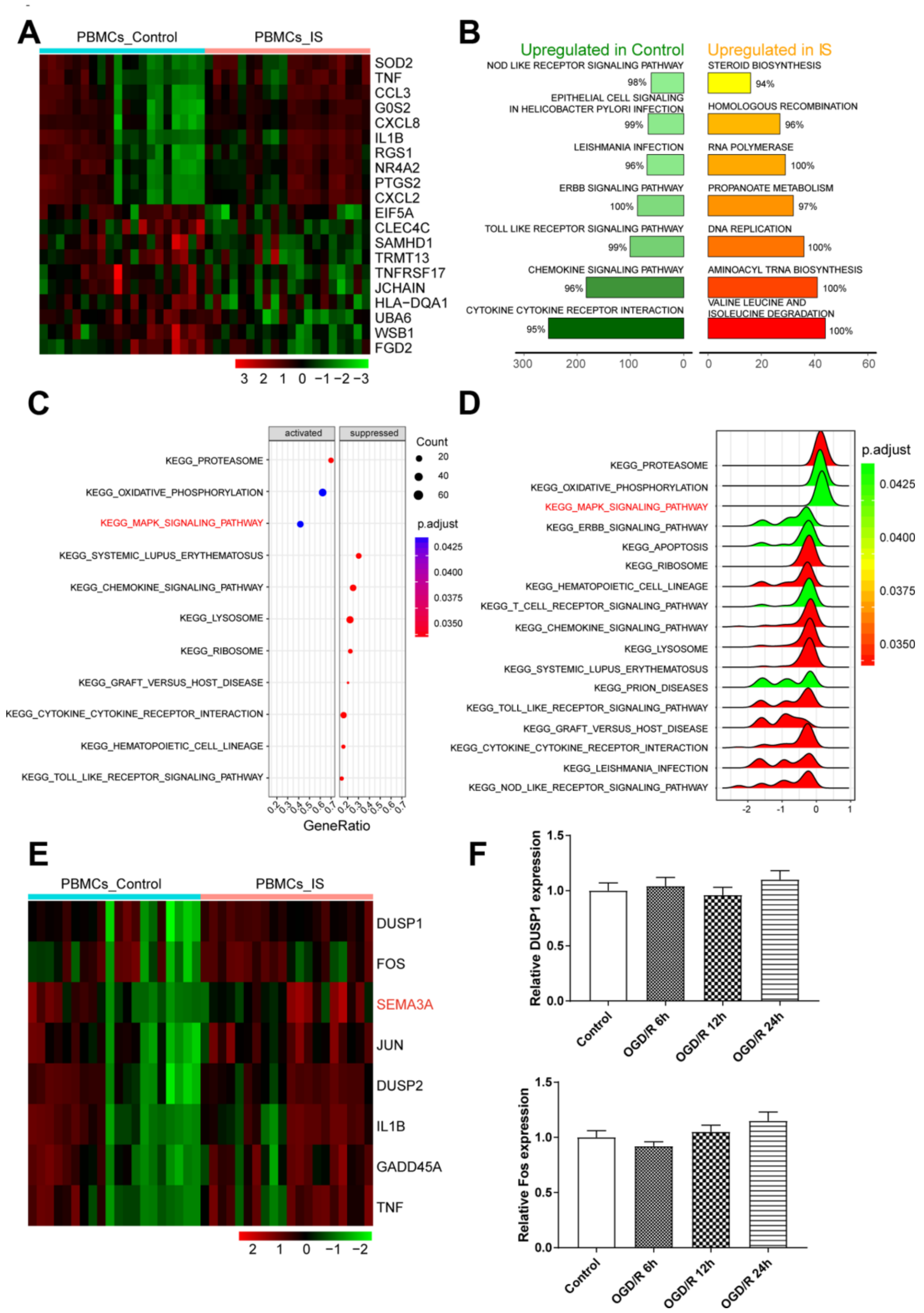

Supplementary Figure 1. *Sema3A* was a highly expressed mRNA related to MAPK signaling pathway in IS. (**A**) The heatmap we achieved during microarray analysis of GSE22255 showed the top 10 differentially expressed mRNAs in IS group. (**B**) 7 most significantly up-regulated Biological pathways in IS or control group were screened out and showed in Rankplot. (**C**, **D**) The dotplot and ridgeplot showed the significantly activated or suppressed pathways in IS. (**E**) The heatmap showed these significantly highly expressed mRNAs including *Sema3A* related to MAPK signaling pathway in IS group. (**F**) qRT-PCR to verify the expression of *DUSP1* and *FOS* in OGD/R cell lines, and no significant differences were detected. IS: ischemic stroke.
