# Supplementary material for: LncRNA MEG3 targeting miR-424-5p via MAPK signaling pathway mediates neuronal apoptosis in ischemic stroke
Source: Aging (Albany NY). 2020 Feb 16;12(4):3156–74. doi: 10.18632/aging.102790 (PMC7066902; doi:10.18632/aging.102790)
Supplement: Supplementary Tables [file aging-12-102790-s001..pdf]

SUPPLEMENTARY TABLES

Supplementary Table 1. Differentially expressed genes between IS cases and controls.

Gene symbol	Log2(Fold Change)	P.Value
EIF5A	-1.1933	0.002099
TNFRSF17	-1.01933	0.002283
UBA6	-0.85768	0.000278
HLA-DQA1	-0.68041	0.008006
WSB1	-0.67531	0.000473
CLEC4C	-0.65535	0.000862
FGD2	-0.64528	0.02306
SAMHD1	-0.64312	0.007638
TRMT13	-0.63659	0.008339
JCHAIN	-0.63134	0.008671
ZCCHC7	-0.60747	0.02924
GPM6B	-0.59836	0.000005.51
ZNF302	-0.59132	0.012926
CELF2	-0.58962	0.033495
CAMK2G	-0.58684	0.019946
IGLL5	-0.58173	0.018621
MZB1	-0.57712	0.004489
ZNF304	-0.57403	0.003304
HSPA1A	-0.5693	0.012828
RUFY2	-0.56391	0.037698
NUP58	-0.56381	0.002709
CENPBD1	-0.55483	0.022392
RNPC3	-0.54977	0.001531
CD48	-0.54693	0.004699
KBTBD7	-0.53894	0.012173
SNX20	-0.52295	0.032681
NBPF1	-0.5223	0.030126
FAM200A	-0.51752	0.018003
ZNF322	-0.51256	0.028601
HEATR3	-0.50571	0.001937
CA2	0.507187	0.040542
ZEB2	0.507211	0.015285
SLC25A37	0.509624	0.044651
STK17B	0.515491	0.020551
ANKRD28	0.520735	0.018214
HDGFRP3	0.524408	0.005869
HLA-DPA1	0.524506	0.041969
GABARAPL1	0.526229	0.038789
OTUD1	0.556794	0.037242
ATF3	0.561912	0.029651
IER2	0.564879	0.003026
CTSG	0.565097	0.017752
BTG2	0.572205	0.016947
GADD45A	0.576533	0.03824
CENPK	0.577414	0.039386

SEMA3A	0.580613	0.040728
WTAP	0.583642	0.018787
BEND2	0.591585	0.015629
RGCC	0.592948	0.019264
OSM	0.608016	0.022611
FAM46C	0.60976	0.043541
MYNN	0.619807	0.002299
DUSP1	0.62327	0.001918
SRSF3	0.652941	0.008371
CDKN1A	0.662769	0.014157
OSR2	0.667399	0.045292
JUNB	0.673469	0.024806
DDIT4	0.684944	0.025786
IVNS1ABP	0.729506	0.033742
EIF1	0.752786	0.003225
NFKBIZ	0.770618	0.011562
ZFP36	0.779854	0.002729
BCL10	0.802735	0.038375
RNF103	0.805624	0.036316
CXCL5	0.827036	0.028031
NFKBIA	0.86851	0.007028
SRGN	0.880942	0.00618
FOS	0.911193	0.01931
NLRP3	0.939853	0.0409
TNFAIP3	0.94393	0.010292
CD69	0.995602	0.008668
JUN	1.004354	0.002012
EGR1	1.044415	0.015669
SOCS3	1.081229	0.024539
CCNL1	1.092057	0.001487
PPP1R15A	1.175647	0.010564
IER3	1.191708	0.004557
SAMSN1	1.22593	0.004521
CD83	1.227051	0.047692
DUSP2	1.231341	0.010523
VIM	1.313405	0.000665
NAMPT	1.32224	0.007202
TMEM107	1.355714	0.0000713
RGS1	1.363352	0.023201
SOD2	1.411694	0.009472
IL1B	1.505815	0.047103
TNF	1.532196	0.005671
NR4A2	1.541275	0.042084
PTGS2	1.732055	0.022318
CCL3	1.892582	0.004771
CXCL2	2.257273	0.020861
G0S2	2.281486	0.004278
CXCL8	2.610594	0.0019

Supplementary Table 2. Differentially expressed non-coding RNAs between IS cases and controls.

Gene symbol	Log2(Fold Change)	P.Value
DLEU2	-0.88707	0.007371
IGHG1	-0.69232	0.018252
HOTAIRM1	-0.56951	0.030839
IGH	-0.56758	0.039072
IGKV4-1	-0.53246	0.020974
IGKC	-0.50469	0.03053
TRAV25	-0.50141	0.048237
MEG3	0.565392	0.002795
TRBV27	0.882075	0.025381
LINC00936	1.109992	0.002261
SNORD3A	1.428808	0.001495
BRE-AS1	1.430751	0.0335

Supplementary Table 3. The RNAi and miRNA mimics and inhibitor sequences for cell transfection.

		Sequences (5′ → 3′)
Si-MEG3 1	Sense	5′- **AACAGCAAAUGGCACAGGAAGAGACGC** -3′
	Antisense	5′- GCGUCUUCCUGUGCCAUUUGCUGUU-3′
Si-MEG3 2	Sense	5′- AACUGGAGGAUGCAGGCUGGAAACA-3′
	Antisense	5′- UGUUUCCAGCCUGCAUCCUCCAGUU -3′
si-*SEMA3A* 1		5′- GGATGGGTCCTCATGCTCAC -3′
si-*SEMA3A* 2		5′- GGAGCAGCAACAAGTGGAA -3′
miR-424-5p mimics		5′- CAGCAGCAAUUCAUGUUUUGGA -3′
miR-424-5p inhibitor		5′- UCCAAAACAUGAAUUGCUGCUG -3′

Supplementary Table 4. The primer sequences for qRT-PCR.

		The primer sequences (5′ → 3′)
MEG3	Forward:	5′- GTGGACAATGGTGTCCAGGC -3′
	Reverse:	5′- TTAACTCAGAGCGGGTCTCC -3′
miR-424-5p	Forward:	5′- ACACTCCAGCTGGGCAGCAGCAATTCATGT -3′
	Reverse:	5′- TGGTGTCGTGGAGTCG -3′
SEMA3A	Forward:	5′- GCCTGCAGAAGAAGGATTCA -3′
	Reverse:	5′- TCAGGTTGGGGTGGTTAATG -3′
GAPDH	Forward:	5′- GTCAACGGATTTGGTCTGTATT -3′
	Reverse:	5′- AGTCTTCTGGGTGGCAGTGAT -3′
U6	Forward:	5′- CTCGCTTCGGCAGCACA -3′
	Reverse:	5′- AACGCTTCACGAATTTGCGT -3′
